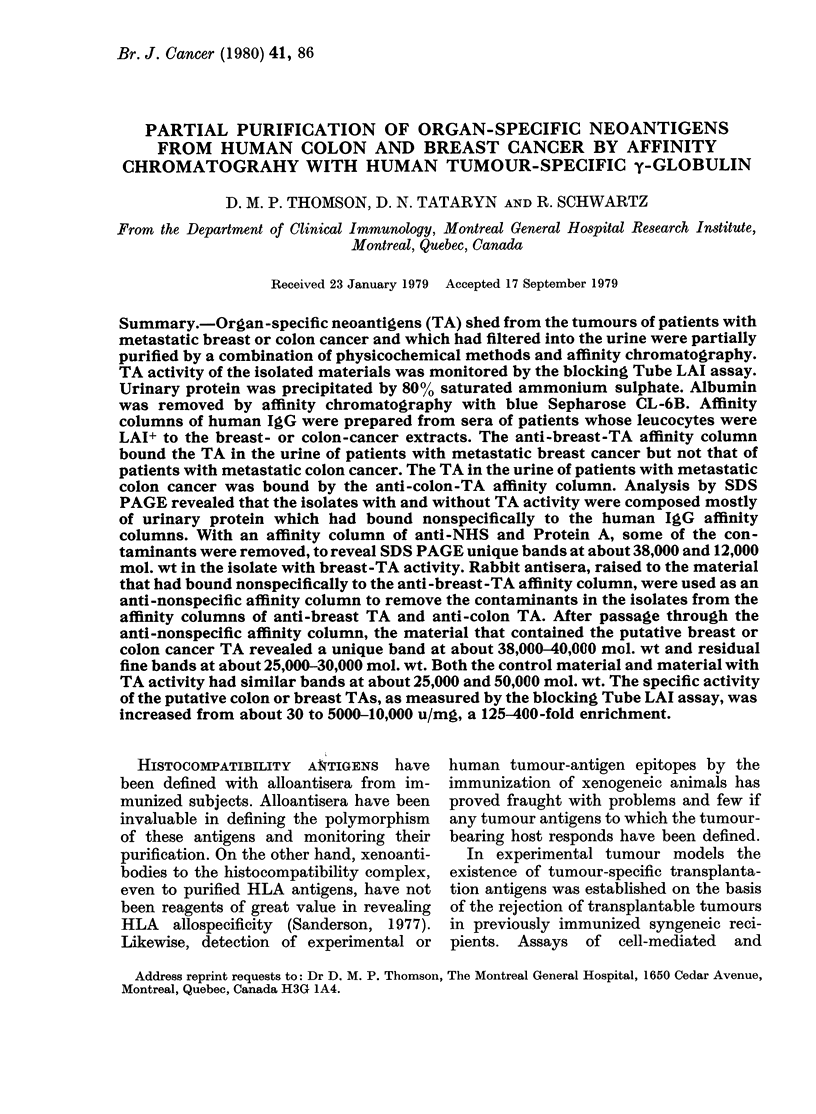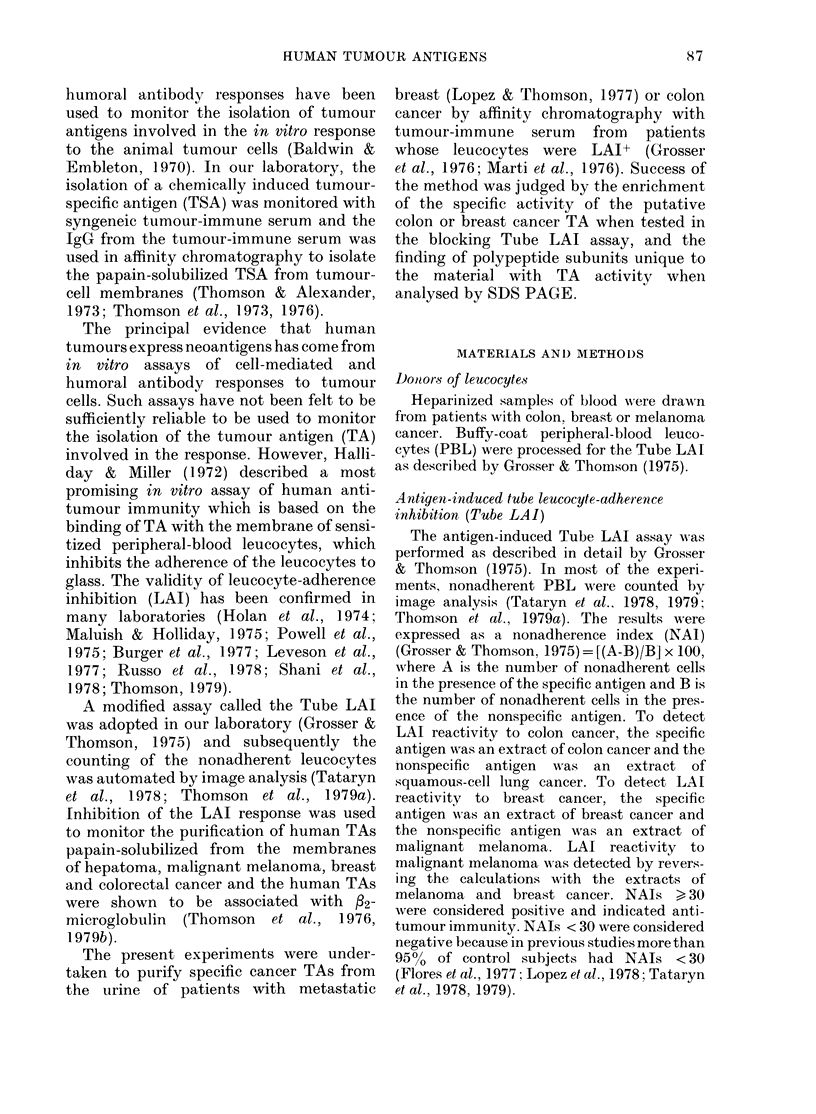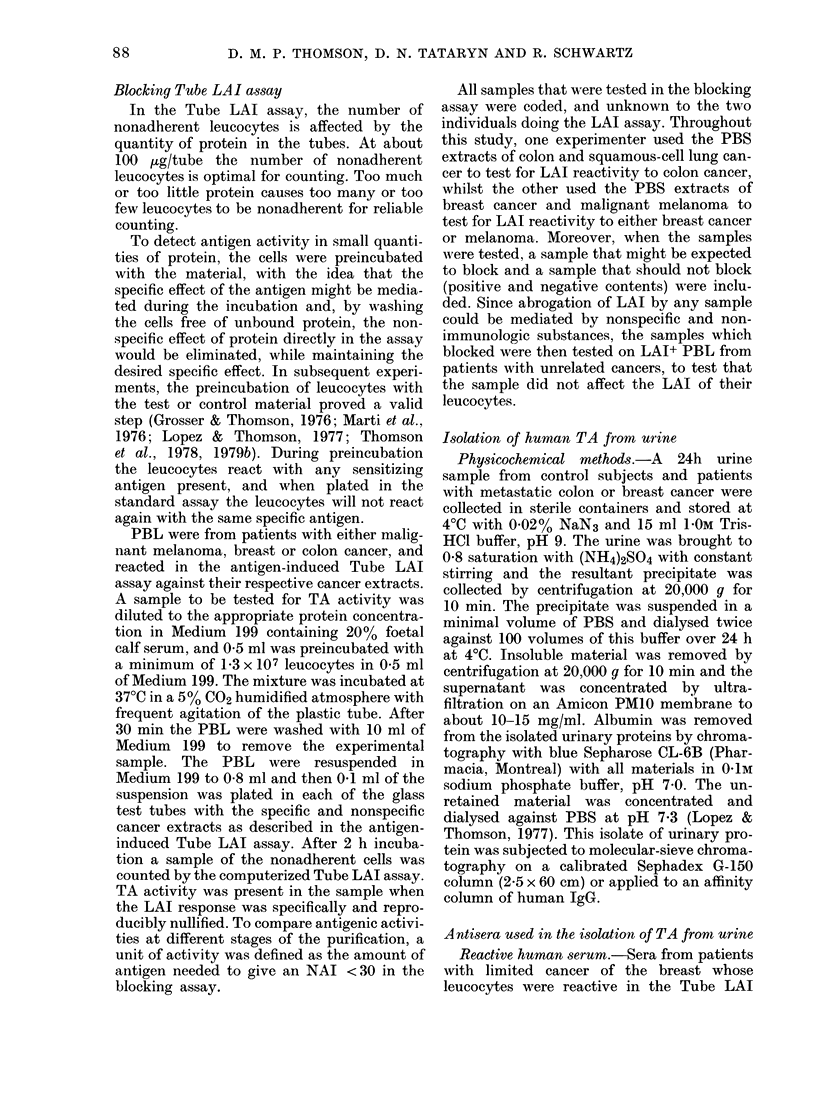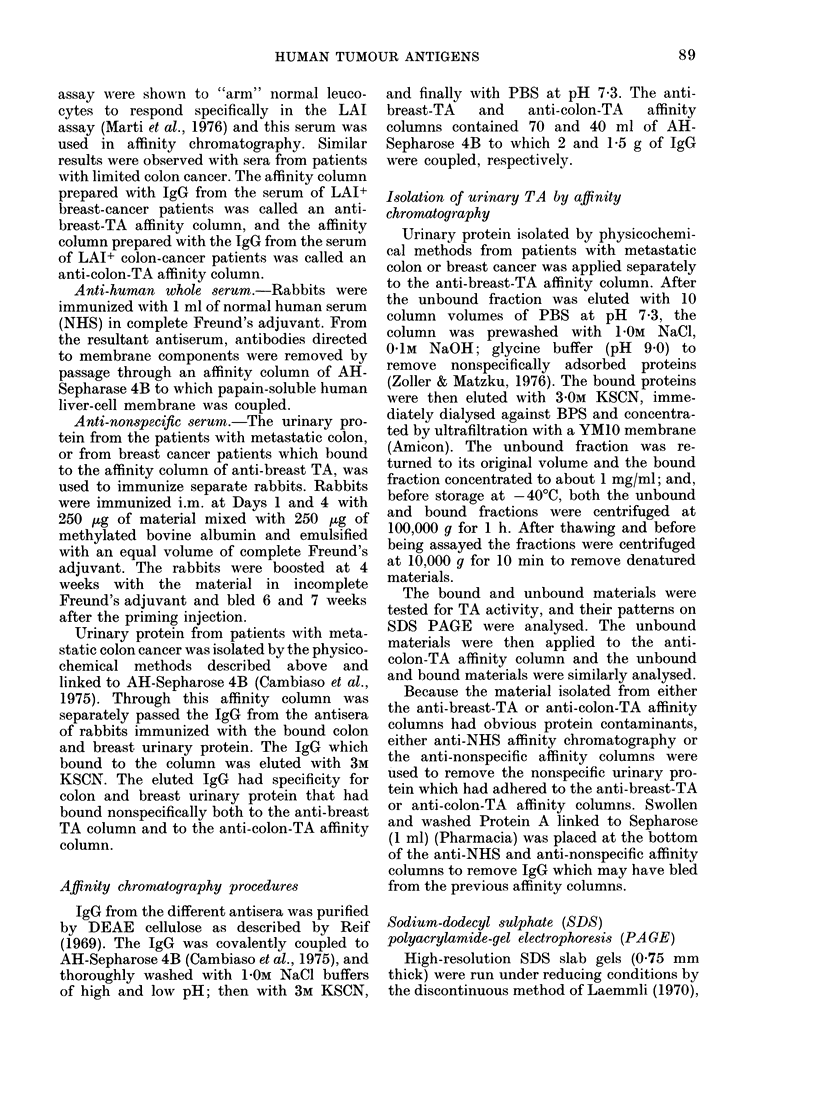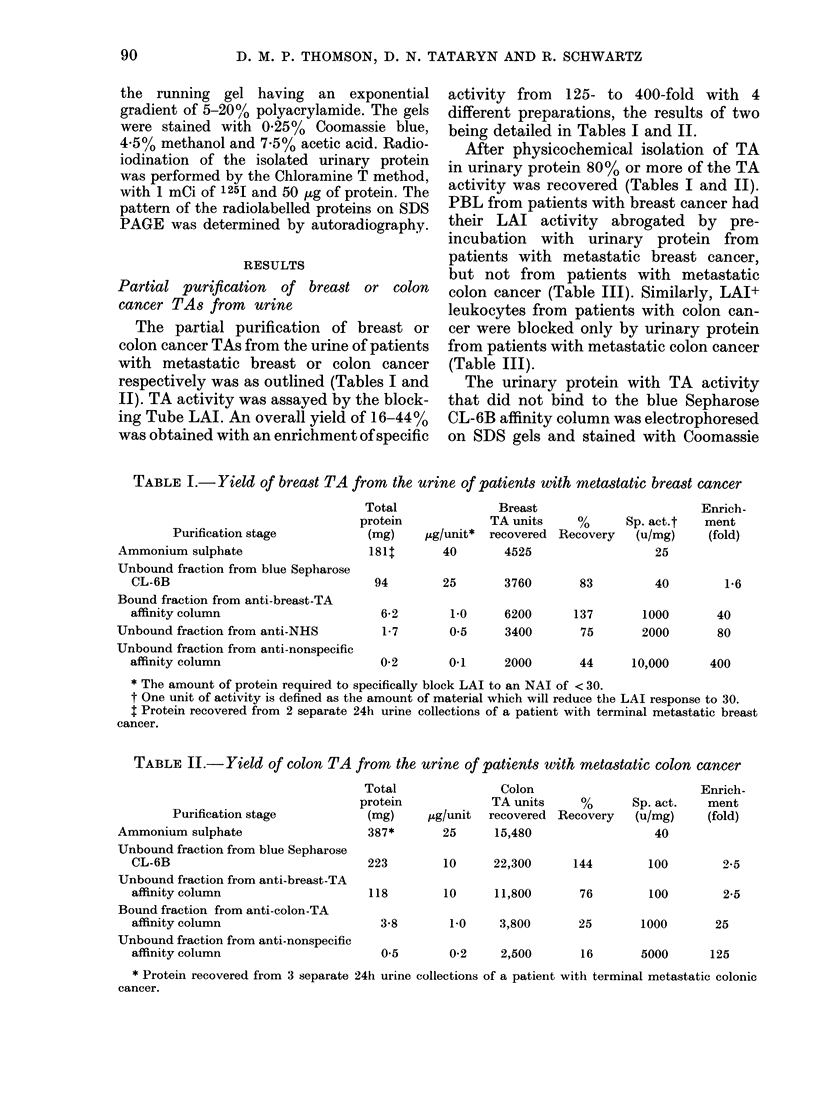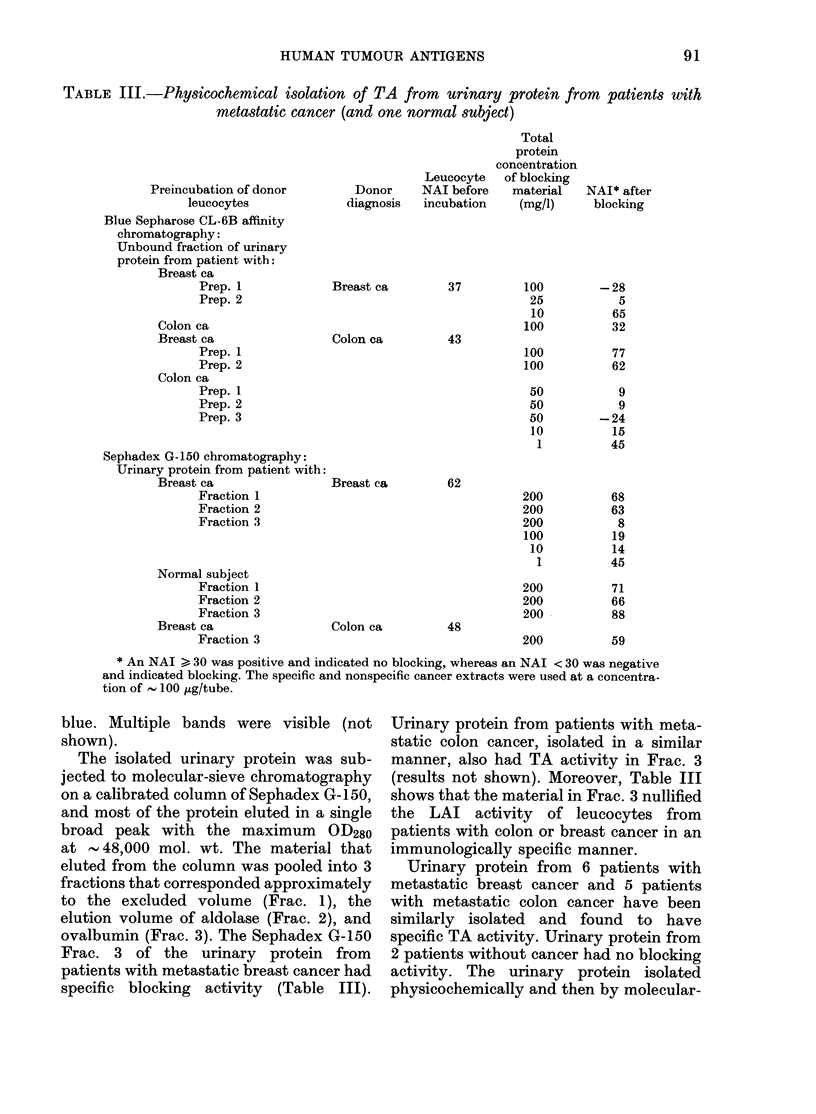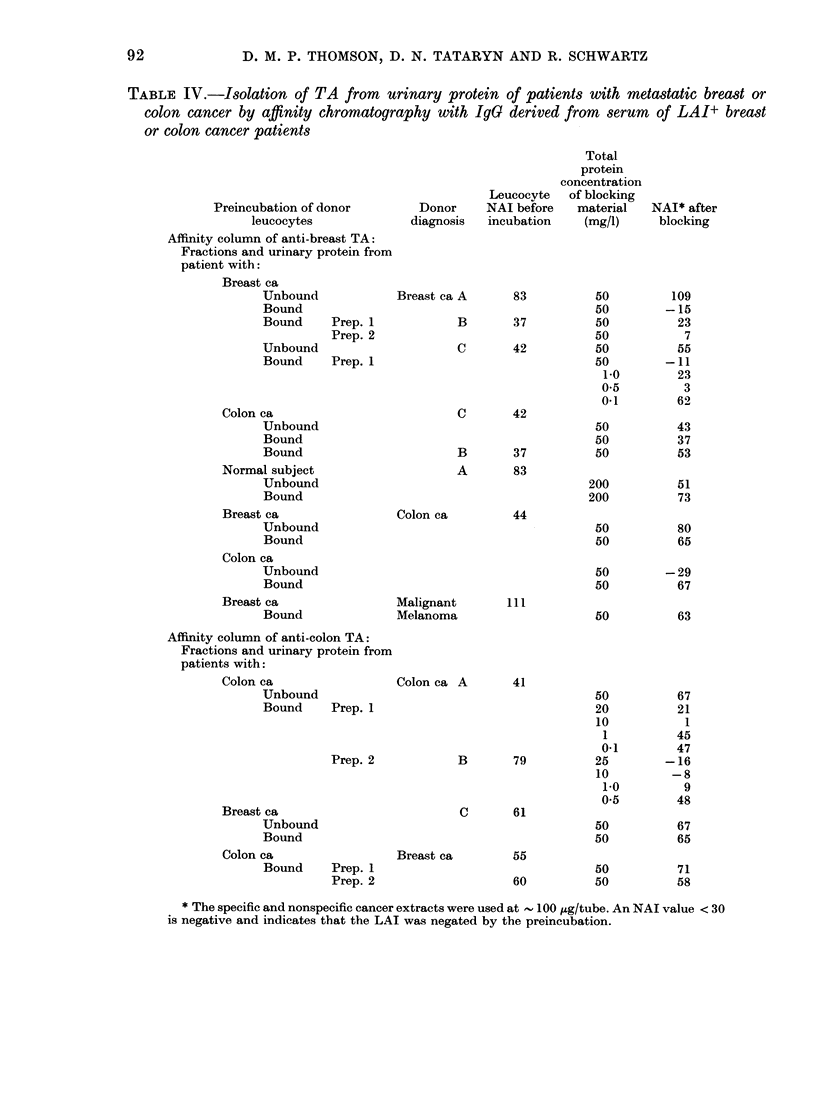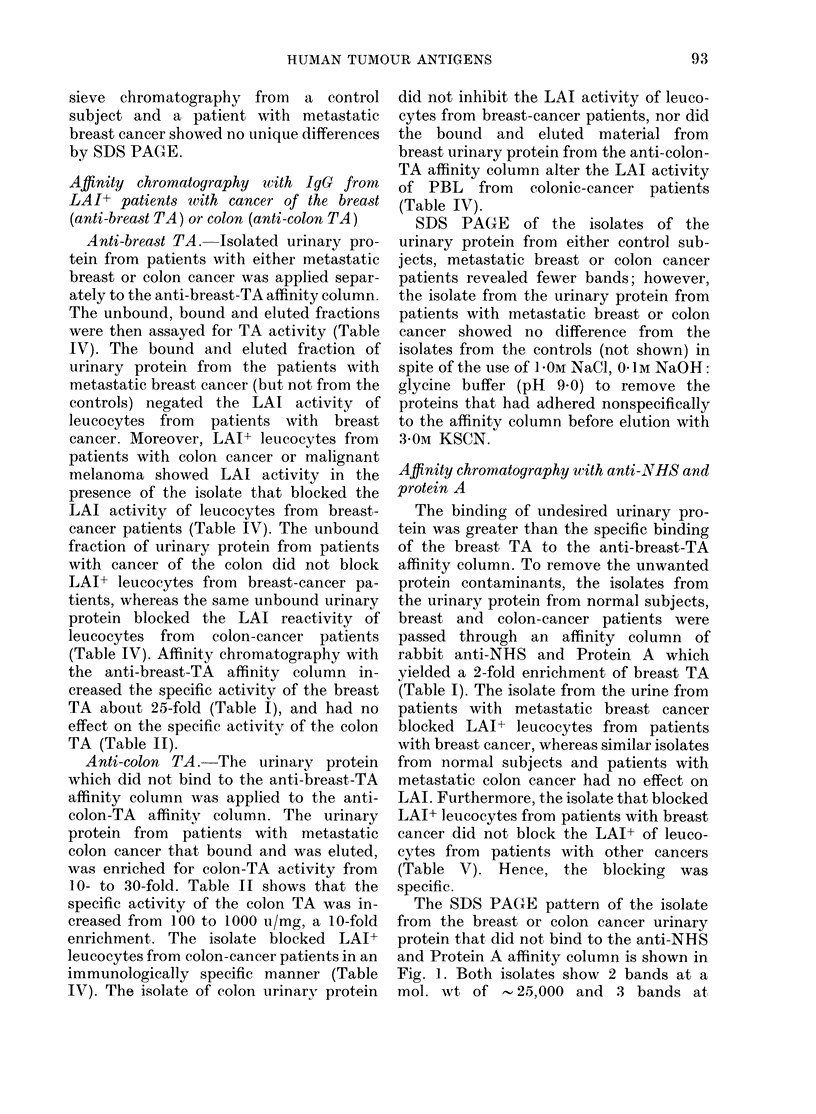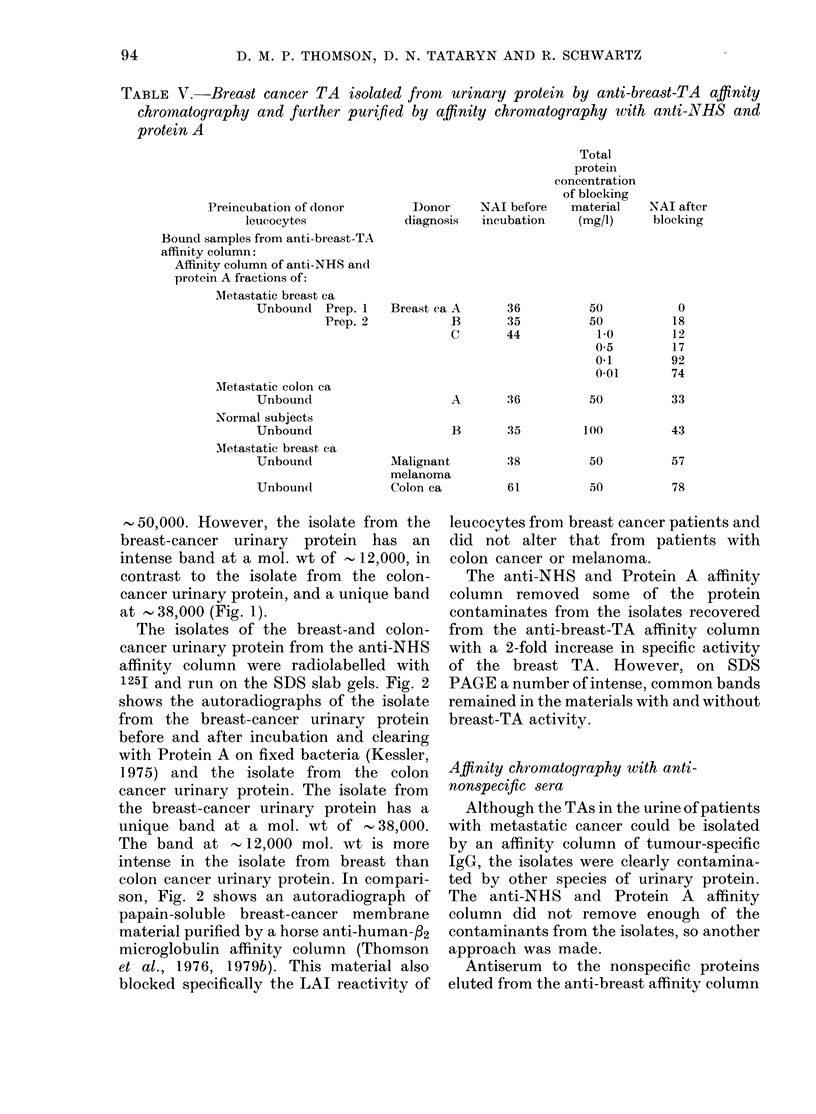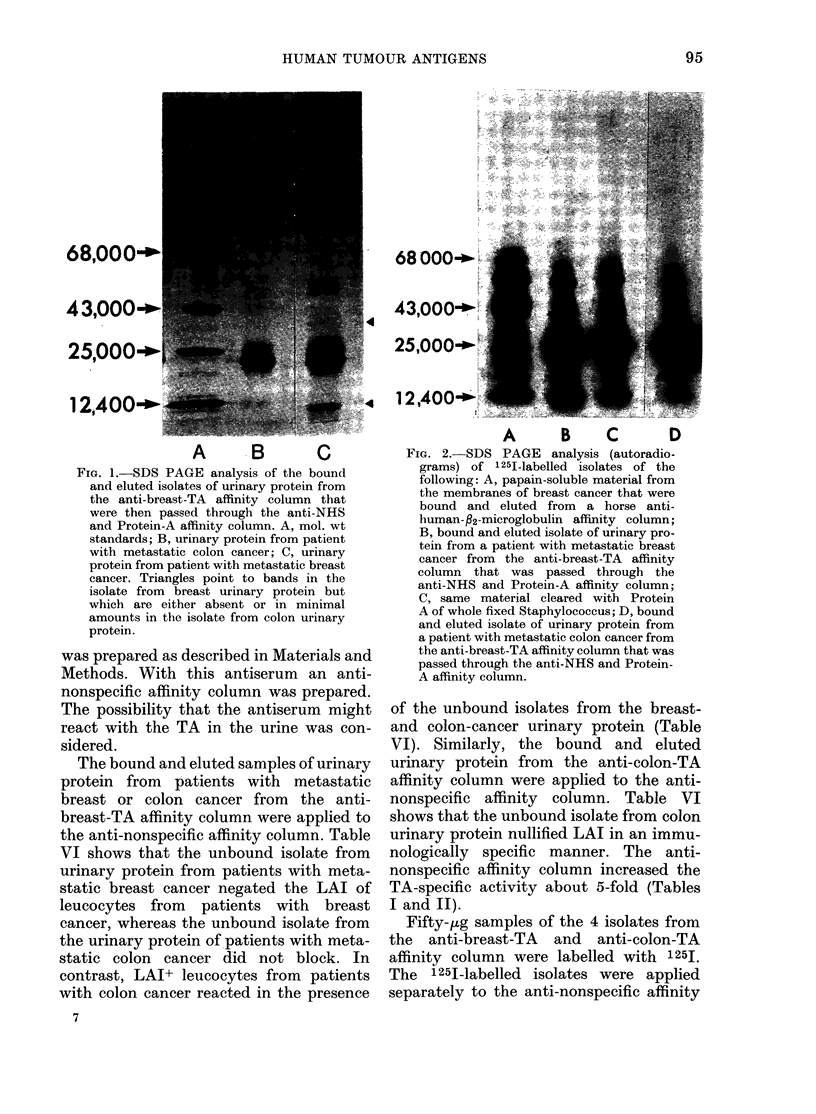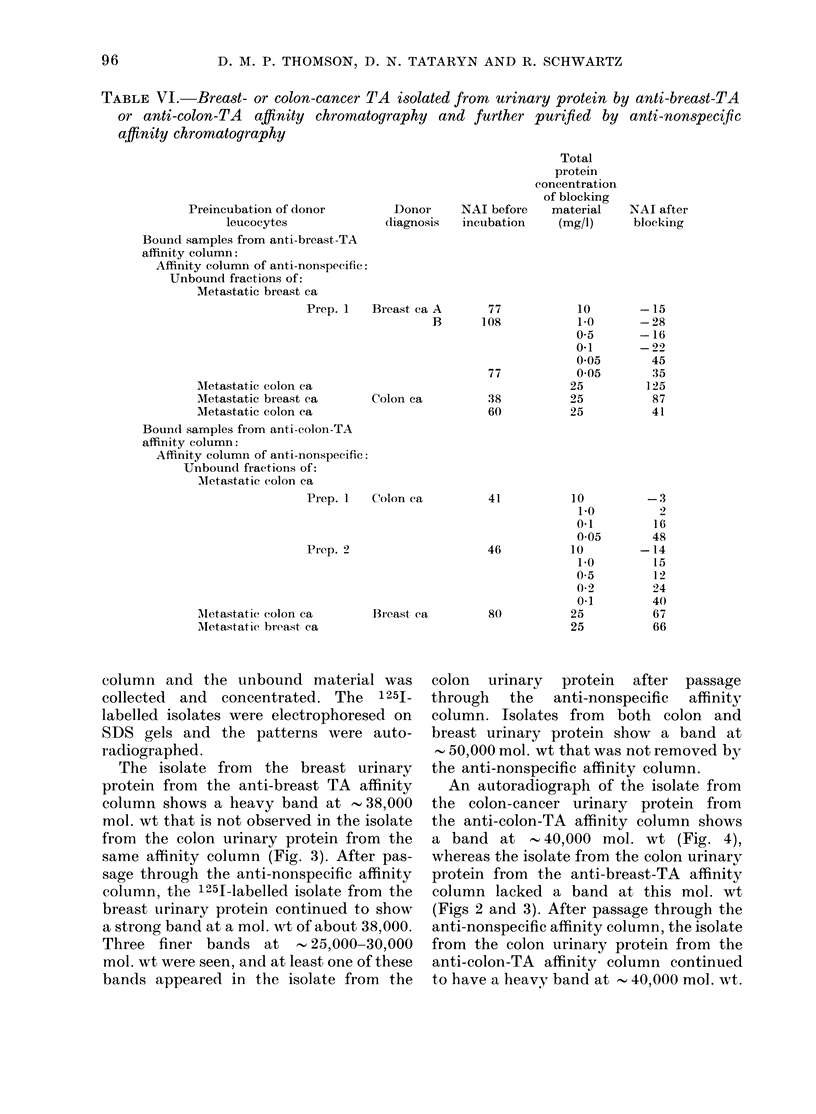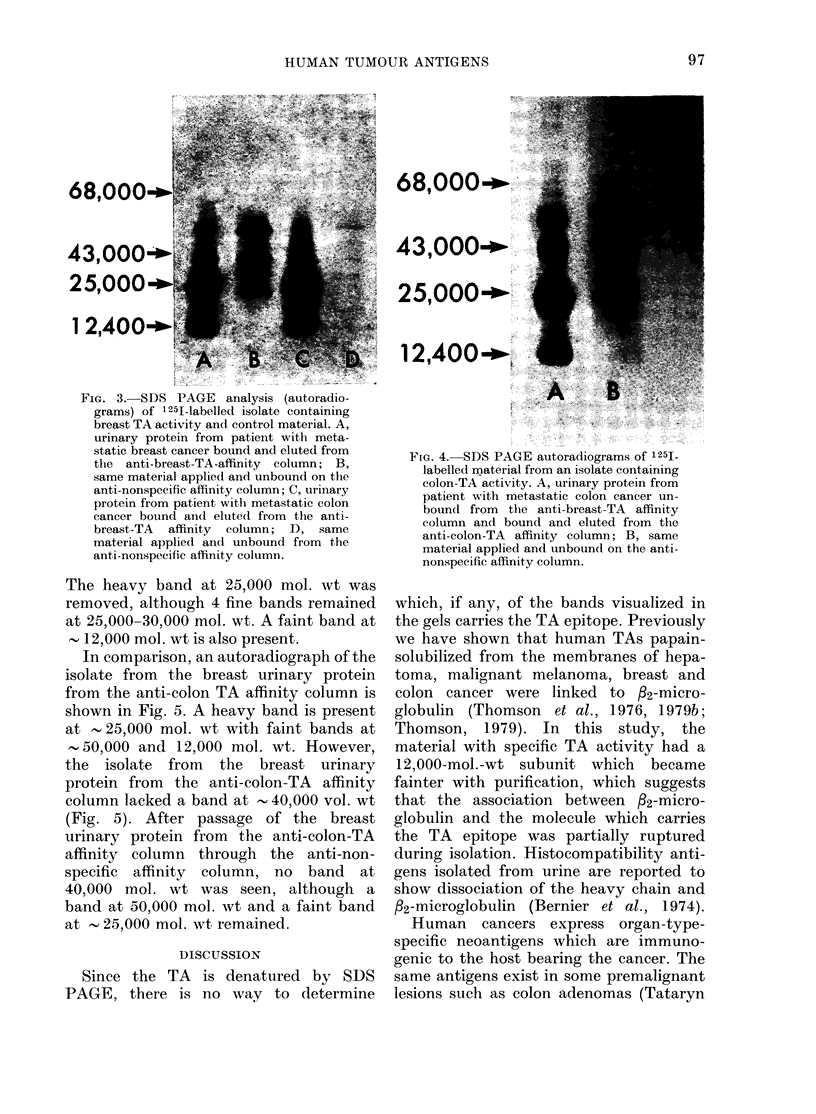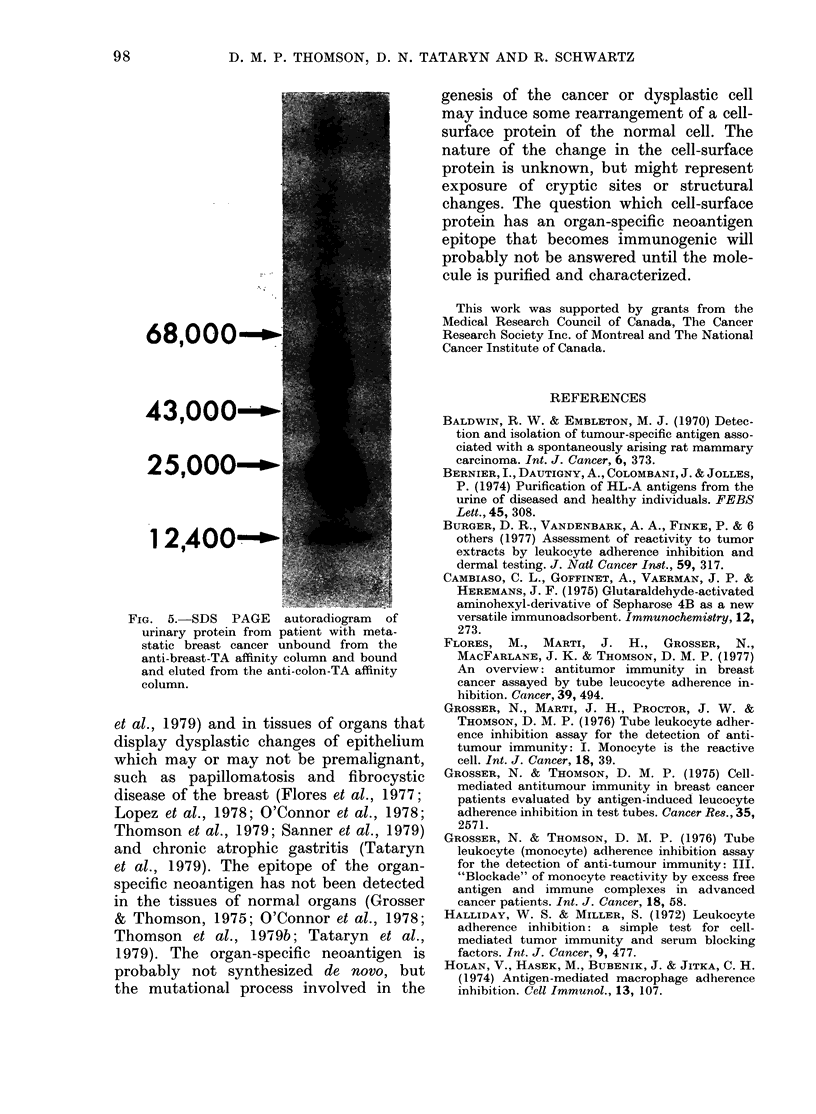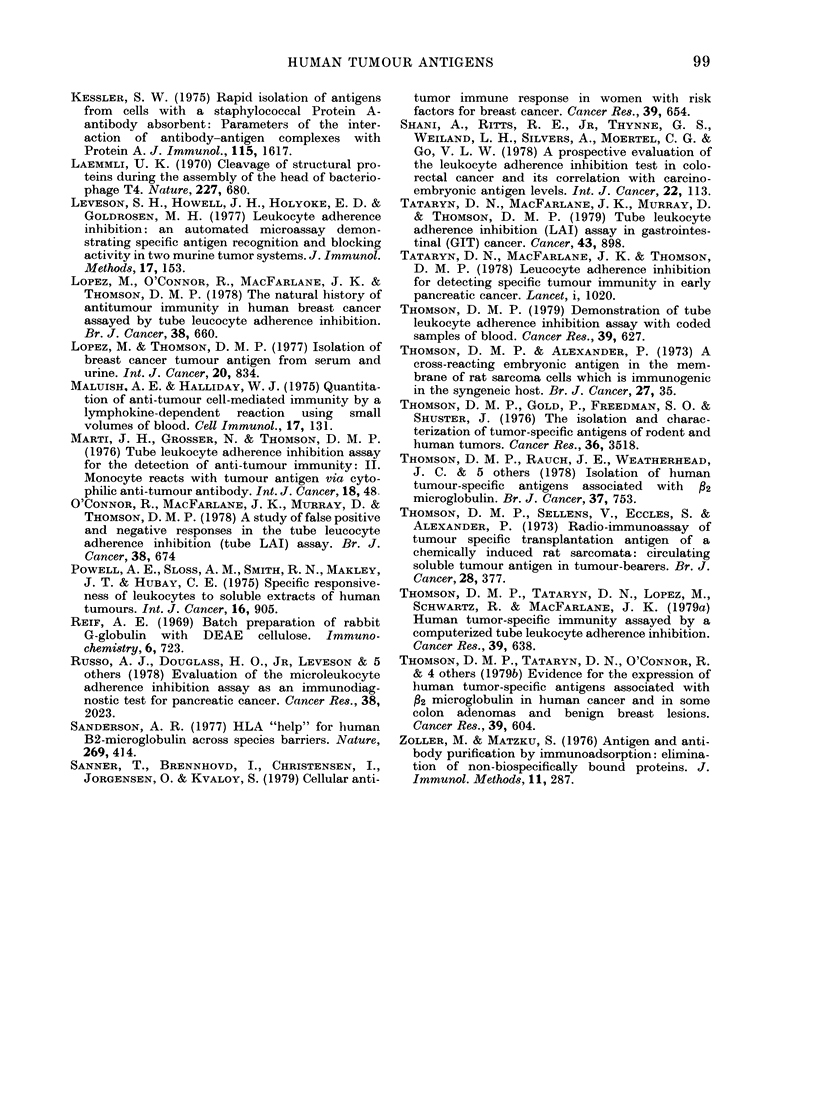# Partial purification of organ-specific neoantigens from human colon and breast cancer by affinity chromatography with human tumour-specific gamma-globulin.

**DOI:** 10.1038/bjc.1980.10

**Published:** 1980-01

**Authors:** D. M. Thomson, D. N. Tataryn, R. Schwartz

## Abstract

**Images:**


*Br. J. Cancer* (1980) **41**, 86

### PARTIAL PURIFICATION OF ORGAN-SPECIFIC NEOANTIGENS FROM HUMAN COLON AND BREAST CANCER BY AFFINITY CHROMATOGRAHY WITH HUMAN TUMOUR-SPECIFIC $\gamma$-GLOBULIN

D. M. P. THOMSON, D. N. TATARYN AND R. SCHWARTZ

*From the Department of Clinical Immunology, Montreal General Hospital Research Institute, Montreal, Quebec, Canada*

Received 23 January 1979 Accepted 17 September 1979

**Summary.—Organ-specific neoantigens (TA) shed from the tumours of patients with metastatic breast or colon cancer and which had filtered into the urine were partially purified by a combination of physicochemical methods and affinity chromatography. TA activity of the isolated materials was monitored by the blocking Tube LAI assay. Urinary protein was precipitated by 80% saturated ammonium sulphate. Albumin was removed by affinity chromatography with blue Sepharose CL-6B. Affinity columns of human IgG were prepared from sera of patients whose leucocytes were LAI$^+$ to the breast- or colon-cancer extracts. The anti-breast-TA affinity column bound the TA in the urine of patients with metastatic breast cancer but not that of patients with metastatic colon cancer. The TA in the urine of patients with metastatic colon cancer was bound by the anti-colon-TA affinity column. Analysis by SDS PAGE revealed that the isolates with and without TA activity were composed mostly of urinary protein which had bound nonspecifically to the human IgG affinity columns. With an affinity column of anti-NHS and Protein A, some of the contaminants were removed, to reveal SDS PAGE unique bands at about 38,000 and 12,000 mol. wt in the isolate with breast-TA activity. Rabbit antisera, raised to the material that had bound nonspecifically to the anti-breast-TA affinity column, were used as an anti-nonspecific affinity column to remove the contaminants in the isolates from the affinity columns of anti-breast TA and anti-colon TA. After passage through the anti-nonspecific affinity column, the material that contained the putative breast or colon cancer TA revealed a unique band at about 38,000–40,000 mol. wt and residual fine bands at about 25,000–30,000 mol. wt. Both the control material and material with TA activity had similar bands at about 25,000 and 50,000 mol. wt. The specific activity of the putative colon or breast TAs, as measured by the blocking Tube LAI assay, was increased from about 30 to 5000–10,000 u/mg, a 125–400-fold enrichment.**

HISTOCOMPATIBILITY ANTIGENS have been defined with alloantisera from immunized subjects. Alloantisera have been invaluable in defining the polymorphism of these antigens and monitoring their purification. On the other hand, xenoantibodies to the histocompatibility complex, even to purified HLA antigens, have not been reagents of great value in revealing HLA allospecificity (Sanderson, 1977). Likewise, detection of experimental or human tumour-antigen epitopes by the immunization of xenogeneic animals has proved fraught with problems and few if any tumour antigens to which the tumour-bearing host responds have been defined.

In experimental tumour models the existence of tumour-specific transplantation antigens was established on the basis of the rejection of transplantable tumours in previously immunized syngeneic recipients. Assays of cell-mediated and

Address reprint requests to: Dr D. M. P. Thomson, The Montreal General Hospital, 1650 Cedar Avenue, Montreal, Quebec, Canada H3G 1A4.

humoral antibody responses have been used to monitor the isolation of tumour antigens involved in the *in vitro* response to the animal tumour cells (Baldwin & Embleton, 1970). In our laboratory, the isolation of a chemically induced tumour-specific antigen (TSA) was monitored with syngeneic tumour-immune serum and the IgG from the tumour-immune serum was used in affinity chromatography to isolate the papain-solubilized TSA from tumour-cell membranes (Thomson & Alexander, 1973; Thomson *et al.*, 1973, 1976).

The principal evidence that human tumours express neoantigens has come from *in vitro* assays of cell-mediated and humoral antibody responses to tumour cells. Such assays have not been felt to be sufficiently reliable to be used to monitor the isolation of the tumour antigen (TA) involved in the response. However, Halliday & Miller (1972) described a most promising *in vitro* assay of human anti-tumour immunity which is based on the binding of TA with the membrane of sensitized peripheral-blood leucocytes, which inhibits the adherence of the leucocytes to glass. The validity of leucocyte-adherence inhibition (LAI) has been confirmed in many laboratories (Holan *et al.*, 1974; Maluish & Holliday, 1975; Powell *et al.*, 1975; Burger *et al.*, 1977; Leveson *et al.*, 1977; Russo *et al.*, 1978; Shani *et al.*, 1978; Thomson, 1979).

A modified assay called the Tube LAI was adopted in our laboratory (Grosser & Thomson, 1975) and subsequently the counting of the nonadherent leucocytes was automated by image analysis (Tataryn *et al.*, 1978; Thomson *et al.*, 1979*a*). Inhibition of the LAI response was used to monitor the purification of human TAs papain-solubilized from the membranes of hepatoma, malignant melanoma, breast and colorectal cancer and the human TAs were shown to be associated with $\beta_2$-microglobulin (Thomson *et al.*, 1976, 1979*b*).

The present experiments were undertaken to purify specific cancer TAs from the urine of patients with metastatic breast (Lopez & Thomson, 1977) or colon cancer by affinity chromatography with tumour-immune serum from patients whose leucocytes were $LAI^+$ (Grosser *et al.*, 1976; Marti *et al.*, 1976). Success of the method was judged by the enrichment of the specific activity of the putative colon or breast cancer TA when tested in the blocking Tube LAI assay, and the finding of polypeptide subunits unique to the material with TA activity when analysed by SDS PAGE.

### MATERIALS AND METHODS

### *Donors of leucocytes*

Heparinized samples of blood were drawn from patients with colon, breast or melanoma cancer. Buffy-coat peripheral-blood leucocytes (PBL) were processed for the Tube LAI as described by Grosser & Thomson (1975).

### *Antigen-induced tube leucocyte-adherence inhibition (Tube LAI)*

The antigen-induced Tube LAI assay was performed as described in detail by Grosser & Thomson (1975). In most of the experiments, nonadherent PBL were counted by image analysis (Tataryn *et al.*, 1978, 1979; Thomson *et al.*, 1979*a*). The results were expressed as a nonadherence index (NAI) (Grosser & Thomson, 1975) $= [(A\text{-}B)/B] \times 100$, where A is the number of nonadherent cells in the presence of the specific antigen and B is the number of nonadherent cells in the presence of the nonspecific antigen. To detect LAI reactivity to colon cancer, the specific antigen was an extract of colon cancer and the nonspecific antigen was an extract of squamous-cell lung cancer. To detect LAI reactivity to breast cancer, the specific antigen was an extract of breast cancer and the nonspecific antigen was an extract of malignant melanoma. LAI reactivity to malignant melanoma was detected by reversing the calculations with the extracts of melanoma and breast cancer. NAIs $\geqslant 30$ were considered positive and indicated anti-tumour immunity. NAIs $< 30$ were considered negative because in previous studies more than 95% of control subjects had NAIs $< 30$ (Flores *et al.*, 1977; Lopez *et al.*, 1978; Tataryn *et al.*, 1978, 1979).

*Blocking Tube LAI assay*

In the Tube LAI assay, the number of nonadherent leucocytes is affected by the quantity of protein in the tubes. At about 100 μg/tube the number of nonadherent leucocytes is optimal for counting. Too much or too little protein causes too many or too few leucocytes to be nonadherent for reliable counting.

To detect antigen activity in small quantities of protein, the cells were preincubated with the material, with the idea that the specific effect of the antigen might be mediated during the incubation and, by washing the cells free of unbound protein, the nonspecific effect of protein directly in the assay would be eliminated, while maintaining the desired specific effect. In subsequent experiments, the preincubation of leucocytes with the test or control material proved a valid step (Grosser & Thomson, 1976; Marti *et al.*, 1976; Lopez & Thomson, 1977; Thomson *et al.*, 1978, 1979*b*). During preincubation the leucocytes react with any sensitizing antigen present, and when plated in the standard assay the leucocytes will not react again with the same specific antigen.

PBL were from patients with either malignant melanoma, breast or colon cancer, and reacted in the antigen-induced Tube LAI assay against their respective cancer extracts. A sample to be tested for TA activity was diluted to the appropriate protein concentration in Medium 199 containing 20% foetal calf serum, and 0·5 ml was preincubated with a minimum of $1{\cdot}3 \times 10^7$ leucocytes in 0·5 ml of Medium 199. The mixture was incubated at 37°C in a 5% $CO_2$ humidified atmosphere with frequent agitation of the plastic tube. After 30 min the PBL were washed with 10 ml of Medium 199 to remove the experimental sample. The PBL were resuspended in Medium 199 to 0·8 ml and then 0·1 ml of the suspension was plated in each of the glass test tubes with the specific and nonspecific cancer extracts as described in the antigen-induced Tube LAI assay. After 2 h incubation a sample of the nonadherent cells was counted by the computerized Tube LAI assay. TA activity was present in the sample when the LAI response was specifically and reproducibly nullified. To compare antigenic activities at different stages of the purification, a unit of activity was defined as the amount of antigen needed to give an NAI $< 30$ in the blocking assay.

All samples that were tested in the blocking assay were coded, and unknown to the two individuals doing the LAI assay. Throughout this study, one experimenter used the PBS extracts of colon and squamous-cell lung cancer to test for LAI reactivity to colon cancer, whilst the other used the PBS extracts of breast cancer and malignant melanoma to test for LAI reactivity to either breast cancer or melanoma. Moreover, when the samples were tested, a sample that might be expected to block and a sample that should not block (positive and negative contents) were included. Since abrogation of LAI by any sample could be mediated by nonspecific and nonimmunologic substances, the samples which blocked were then tested on $LAI^+$ PBL from patients with unrelated cancers, to test that the sample did not affect the LAI of their leucocytes.

*Isolation of human TA from urine*

*Physicochemical methods.*—A 24h urine sample from control subjects and patients with metastatic colon or breast cancer were collected in sterile containers and stored at 4°C with 0·02% $NaN_3$ and 15 ml 1·0M Tris-HCl buffer, pH 9. The urine was brought to 0·8 saturation with $(NH_4)_2SO_4$ with constant stirring and the resultant precipitate was collected by centrifugation at 20,000 *g* for 10 min. The precipitate was suspended in a minimal volume of PBS and dialysed twice against 100 volumes of this buffer over 24 h at 4°C. Insoluble material was removed by centrifugation at 20,000 *g* for 10 min and the supernatant was concentrated by ultrafiltration on an Amicon PM10 membrane to about 10–15 mg/ml. Albumin was removed from the isolated urinary proteins by chromatography with blue Sepharose CL-6B (Pharmacia, Montreal) with all materials in 0·1M sodium phosphate buffer, pH 7·0. The unretained material was concentrated and dialysed against PBS at pH 7·3 (Lopez & Thomson, 1977). This isolate of urinary protein was subjected to molecular-sieve chromatography on a calibrated Sephadex G-150 column (2·5 × 60 cm) or applied to an affinity column of human IgG.

*Antisera used in the isolation of TA from urine*

*Reactive human serum.*—Sera from patients with limited cancer of the breast whose leucocytes were reactive in the Tube LAI

assay were shown to "arm" normal leucocytes to respond specifically in the LAI assay (Marti *et al.*, 1976) and this serum was used in affinity chromatography. Similar results were observed with sera from patients with limited colon cancer. The affinity column prepared with IgG from the serum of LAI+ breast-cancer patients was called an anti-breast-TA affinity column, and the affinity column prepared with the IgG from the serum of LAI+ colon-cancer patients was called an anti-colon-TA affinity column.

*Anti-human whole serum.*—Rabbits were immunized with 1 ml of normal human serum (NHS) in complete Freund's adjuvant. From the resultant antiserum, antibodies directed to membrane components were removed by passage through an affinity column of AH-Sepharase 4B to which papain-soluble human liver-cell membrane was coupled.

*Anti-nonspecific serum.*—The urinary protein from the patients with metastatic colon, or from breast cancer patients which bound to the affinity column of anti-breast TA, was used to immunize separate rabbits. Rabbits were immunized i.m. at Days 1 and 4 with 250 μg of material mixed with 250 μg of methylated bovine albumin and emulsified with an equal volume of complete Freund's adjuvant. The rabbits were boosted at 4 weeks with the material in incomplete Freund's adjuvant and bled 6 and 7 weeks after the priming injection.

Urinary protein from patients with metastatic colon cancer was isolated by the physicochemical methods described above and linked to AH-Sepharose 4B (Cambiaso *et al.*, 1975). Through this affinity column was separately passed the IgG from the antisera of rabbits immunized with the bound colon and breast urinary protein. The IgG which bound to the column was eluted with 3M KSCN. The eluted IgG had specificity for colon and breast urinary protein that had bound nonspecifically both to the anti-breast TA column and to the anti-colon-TA affinity column.

*Affinity chromatography procedures*

IgG from the different antisera was purified by DEAE cellulose as described by Reif (1969). The IgG was covalently coupled to AH-Sepharose 4B (Cambiaso *et al.*, 1975), and thoroughly washed with 1·0M NaCl buffers of high and low pH; then with 3M KSCN, and finally with PBS at pH 7·3. The anti-breast-TA and anti-colon-TA affinity columns contained 70 and 40 ml of AH-Sepharose 4B to which 2 and 1·5 g of IgG were coupled, respectively.

*Isolation of urinary TA by affinity chromatography*

Urinary protein isolated by physicochemical methods from patients with metastatic colon or breast cancer was applied separately to the anti-breast-TA affinity column. After the unbound fraction was eluted with 10 column volumes of PBS at pH 7·3, the column was prewashed with 1·0M NaCl, 0·1M NaOH; glycine buffer (pH 9·0) to remove nonspecifically adsorbed proteins (Zoller & Matzku, 1976). The bound proteins were then eluted with 3·0M KSCN, immediately dialysed against BPS and concentrated by ultrafiltration with a YM10 membrane (Amicon). The unbound fraction was returned to its original volume and the bound fraction concentrated to about 1 mg/ml; and, before storage at −40°C, both the unbound and bound fractions were centrifuged at 100,000 *g* for 1 h. After thawing and before being assayed the fractions were centrifuged at 10,000 *g* for 10 min to remove denatured materials.

The bound and unbound materials were tested for TA activity, and their patterns on SDS PAGE were analysed. The unbound materials were then applied to the anti-colon-TA affinity column and the unbound and bound materials were similarly analysed.

Because the material isolated from either the anti-breast-TA or anti-colon-TA affinity columns had obvious protein contaminants, either anti-NHS affinity chromatography or the anti-nonspecific affinity columns were used to remove the nonspecific urinary protein which had adhered to the anti-breast-TA or anti-colon-TA affinity columns. Swollen and washed Protein A linked to Sepharose (1 ml) (Pharmacia) was placed at the bottom of the anti-NHS and anti-nonspecific affinity columns to remove IgG which may have bled from the previous affinity columns.

*Sodium-dodecyl sulphate (SDS) polyacrylamide-gel electrophoresis (PAGE)*

High-resolution SDS slab gels (0·75 mm thick) were run under reducing conditions by the discontinuous method of Laemmli (1970), the running gel having an exponential gradient of 5–20% polyacrylamide. The gels were stained with 0·25% Coomassie blue, 4·5% methanol and 7·5% acetic acid. Radioiodination of the isolated urinary protein was performed by the Chloramine T method, with 1 mCi of $^{125}$I and 50 μg of protein. The pattern of the radiolabelled proteins on SDS PAGE was determined by autoradiography.

### RESULTS

### *Partial purification of breast or colon cancer TAs from urine*

The partial purification of breast or colon cancer TAs from the urine of patients with metastatic breast or colon cancer respectively was as outlined (Tables I and II). TA activity was assayed by the blocking Tube LAI. An overall yield of 16–44% was obtained with an enrichment of specific activity from 125- to 400-fold with 4 different preparations, the results of two being detailed in Tables I and II.

After physicochemical isolation of TA in urinary protein 80% or more of the TA activity was recovered (Tables I and II). PBL from patients with breast cancer had their LAI activity abrogated by preincubation with urinary protein from patients with metastatic breast cancer, but not from patients with metastatic colon cancer (Table III). Similarly, LAI$^+$ leukocytes from patients with colon cancer were blocked only by urinary protein from patients with metastatic colon cancer (Table III).

The urinary protein with TA activity that did not bind to the blue Sepharose CL-6B affinity column was electrophoresed on SDS gels and stained with Coomassie

TABLE I.—*Yield of breast TA from the urine of patients with metastatic breast cancer*

| Purification stage | Total protein (mg) | μg/unit* | Breast TA units recovered | % Recovery | Sp. act.† (u/mg) | Enrichment (fold) |
|---|---|---|---|---|---|---|
| Ammonium sulphate | 181‡ | 40 | 4525 | | 25 | |
| Unbound fraction from blue Sepharose CL-6B | 94 | 25 | 3760 | 83 | 40 | 1·6 |
| Bound fraction from anti-breast-TA affinity column | 6·2 | 1·0 | 6200 | 137 | 1000 | 40 |
| Unbound fraction from anti-NHS | 1·7 | 0·5 | 3400 | 75 | 2000 | 80 |
| Unbound fraction from anti-nonspecific affinity column | 0·2 | 0·1 | 2000 | 44 | 10,000 | 400 |

* The amount of protein required to specifically block LAI to an NAI of < 30.

† One unit of activity is defined as the amount of material which will reduce the LAI response to 30.

‡ Protein recovered from 2 separate 24h urine collections of a patient with terminal metastatic breast cancer.

TABLE II.—*Yield of colon TA from the urine of patients with metastatic colon cancer*

| Purification stage | Total protein (mg) | μg/unit | Colon TA units recovered | % Recovery | Sp. act. (u/mg) | Enrichment (fold) |
|---|---|---|---|---|---|---|
| Ammonium sulphate | 387* | 25 | 15,480 | | 40 | |
| Unbound fraction from blue Sepharose CL-6B | 223 | 10 | 22,300 | 144 | 100 | 2·5 |
| Unbound fraction from anti-breast-TA affinity column | 118 | 10 | 11,800 | 76 | 100 | 2·5 |
| Bound fraction from anti-colon-TA affinity column | 3·8 | 1·0 | 3,800 | 25 | 1000 | 25 |
| Unbound fraction from anti-nonspecific affinity column | 0·5 | 0·2 | 2,500 | 16 | 5000 | 125 |

* Protein recovered from 3 separate 24h urine collections of a patient with terminal metastatic colonic cancer.

TABLE III.—*Physicochemical isolation of TA from urinary protein from patients with metastatic cancer (and one normal subject)*

| Preincubation of donor leucocytes | Donor diagnosis | Leucocyte NAI before incubation | Total protein concentration of blocking material (mg/l) | NAI* after blocking |
|---|---|---|---|---|
| Blue Sepharose CL-6B affinity chromatography: | | | | |
| Unbound fraction of urinary protein from patient with: | | | | |
| Breast ca | | | | |
| Prep. 1 | Breast ca | 37 | 100 | −28 |
| Prep. 2 | | | 25 | 5 |
| | | | 10 | 65 |
| Colon ca | | | 100 | 32 |
| Breast ca | Colon ca | 43 | | |
| Prep. 1 | | | 100 | 77 |
| Prep. 2 | | | 100 | 62 |
| Colon ca | | | | |
| Prep. 1 | | | 50 | 9 |
| Prep. 2 | | | 50 | 9 |
| Prep. 3 | | | 50 | −24 |
| | | | 10 | 15 |
| | | | 1 | 45 |
| Sephadex G-150 chromatography: | | | | |
| Urinary protein from patient with: | | | | |
| Breast ca | Breast ca | 62 | | |
| Fraction 1 | | | 200 | 68 |
| Fraction 2 | | | 200 | 63 |
| Fraction 3 | | | 200 | 8 |
| | | | 100 | 19 |
| | | | 10 | 14 |
| | | | 1 | 45 |
| Normal subject | | | | |
| Fraction 1 | | | 200 | 71 |
| Fraction 2 | | | 200 | 66 |
| Fraction 3 | | | 200 | 88 |
| Breast ca | Colon ca | 48 | | |
| Fraction 3 | | | 200 | 59 |

* An NAI $\geqslant 30$ was positive and indicated no blocking, whereas an NAI $< 30$ was negative and indicated blocking. The specific and nonspecific cancer extracts were used at a concentration of $\sim 100$ $\mu$g/tube.

blue. Multiple bands were visible (not shown).

The isolated urinary protein was subjected to molecular-sieve chromatography on a calibrated column of Sephadex G-150, and most of the protein eluted in a single broad peak with the maximum $OD_{280}$ at $\sim$48,000 mol. wt. The material that eluted from the column was pooled into 3 fractions that corresponded approximately to the excluded volume (Frac. 1), the elution volume of aldolase (Frac. 2), and ovalbumin (Frac. 3). The Sephadex G-150 Frac. 3 of the urinary protein from patients with metastatic breast cancer had specific blocking activity (Table III). Urinary protein from patients with metastatic colon cancer, isolated in a similar manner, also had TA activity in Frac. 3 (results not shown). Moreover, Table III shows that the material in Frac. 3 nullified the LAI activity of leucocytes from patients with colon or breast cancer in an immunologically specific manner.

Urinary protein from 6 patients with metastatic breast cancer and 5 patients with metastatic colon cancer have been similarly isolated and found to have specific TA activity. Urinary protein from 2 patients without cancer had no blocking activity. The urinary protein isolated physicochemically and then by molecular-

TABLE IV.—*Isolation of TA from urinary protein of patients with metastatic breast or colon cancer by affinity chromatography with IgG derived from serum of $LAI^+$ breast or colon cancer patients*

| Preincubation of donor leucocytes | Donor diagnosis | Leucocyte NAI before incubation | Total protein concentration of blocking material (mg/l) | NAI* after blocking |
|---|---|---|---|---|
| Affinity column of anti-breast TA: Fractions and urinary protein from patient with: | | | | |
| Breast ca | | | | |
| Unbound | Breast ca A | 83 | 50 | 109 |
| Bound | | | 50 | −15 |
| Bound Prep. 1 | B | 37 | 50 | 23 |
| Prep. 2 | | | 50 | 7 |
| Unbound | C | 42 | 50 | 55 |
| Bound Prep. 1 | | | 50 | −11 |
| | | | 1·0 | 23 |
| | | | 0·5 | 3 |
| | | | 0·1 | 62 |
| Colon ca | C | 42 | | |
| Unbound | | | 50 | 43 |
| Bound | | | 50 | 37 |
| Bound | B | 37 | 50 | 53 |
| Normal subject | A | 83 | | |
| Unbound | | | 200 | 51 |
| Bound | | | 200 | 73 |
| Breast ca | Colon ca | 44 | | |
| Unbound | | | 50 | 80 |
| Bound | | | 50 | 65 |
| Colon ca | | | | |
| Unbound | | | 50 | −29 |
| Bound | | | 50 | 67 |
| Breast ca | Malignant | 111 | | |
| Bound | Melanoma | | 50 | 63 |
| Affinity column of anti-colon TA: Fractions and urinary protein from patients with: | | | | |
| Colon ca | Colon ca A | 41 | | |
| Unbound | | | 50 | 67 |
| Bound Prep. 1 | | | 20 | 21 |
| | | | 10 | 1 |
| | | | 1 | 45 |
| | | | 0·1 | 47 |
| Prep. 2 | B | 79 | 25 | −16 |
| | | | 10 | −8 |
| | | | 1·0 | 9 |
| | | | 0·5 | 48 |
| Breast ca | C | 61 | | |
| Unbound | | | 50 | 67 |
| Bound | | | 50 | 65 |
| Colon ca | Breast ca | 55 | | |
| Bound Prep. 1 | | | 50 | 71 |
| Prep. 2 | | 60 | 50 | 58 |

* The specific and nonspecific cancer extracts were used at ~100 μg/tube. An NAI value < 30 is negative and indicates that the LAI was negated by the preincubation.

sieve chromatography from a control subject and a patient with metastatic breast cancer showed no unique differences by SDS PAGE.

*Affinity chromatography with IgG from LAI$^+$ patients with cancer of the breast (anti-breast TA) or colon (anti-colon TA)*

*Anti-breast TA.*—Isolated urinary protein from patients with either metastatic breast or colon cancer was applied separately to the anti-breast-TA affinity column. The unbound, bound and eluted fractions were then assayed for TA activity (Table IV). The bound and eluted fraction of urinary protein from the patients with metastatic breast cancer (but not from the controls) negated the LAI activity of leucocytes from patients with breast cancer. Moreover, LAI$^+$ leucocytes from patients with colon cancer or malignant melanoma showed LAI activity in the presence of the isolate that blocked the LAI activity of leucocytes from breast-cancer patients (Table IV). The unbound fraction of urinary protein from patients with cancer of the colon did not block LAI$^+$ leucocytes from breast-cancer patients, whereas the same unbound urinary protein blocked the LAI reactivity of leucocytes from colon-cancer patients (Table IV). Affinity chromatography with the anti-breast-TA affinity column increased the specific activity of the breast TA about 25-fold (Table I), and had no effect on the specific activity of the colon TA (Table II).

*Anti-colon TA.*—The urinary protein which did not bind to the anti-breast-TA affinity column was applied to the anti-colon-TA affinity column. The urinary protein from patients with metastatic colon cancer that bound and was eluted, was enriched for colon-TA activity from 10- to 30-fold. Table II shows that the specific activity of the colon TA was increased from 100 to 1000 u/mg, a 10-fold enrichment. The isolate blocked LAI$^+$ leucocytes from colon-cancer patients in an immunologically specific manner (Table IV). The isolate of colon urinary protein did not inhibit the LAI activity of leucocytes from breast-cancer patients, nor did the bound and eluted material from breast urinary protein from the anti-colon-TA affinity column alter the LAI activity of PBL from colonic-cancer patients (Table IV).

SDS PAGE of the isolates of the urinary protein from either control subjects, metastatic breast or colon cancer patients revealed fewer bands; however, the isolate from the urinary protein from patients with metastatic breast or colon cancer showed no difference from the isolates from the controls (not shown) in spite of the use of 1·0M NaCl, 0·1M NaOH: glycine buffer (pH 9·0) to remove the proteins that had adhered nonspecifically to the affinity column before elution with 3·0M KSCN.

*Affinity chromatography with anti-NHS and protein A*

The binding of undesired urinary protein was greater than the specific binding of the breast TA to the anti-breast-TA affinity column. To remove the unwanted protein contaminants, the isolates from the urinary protein from normal subjects, breast and colon-cancer patients were passed through an affinity column of rabbit anti-NHS and Protein A which yielded a 2-fold enrichment of breast TA (Table I). The isolate from the urine from patients with metastatic breast cancer blocked LAI$^+$ leucocytes from patients with breast cancer, whereas similar isolates from normal subjects and patients with metastatic colon cancer had no effect on LAI. Furthermore, the isolate that blocked LAI$^+$ leucocytes from patients with breast cancer did not block the LAI$^+$ of leucocytes from patients with other cancers (Table V). Hence, the blocking was specific.

The SDS PAGE pattern of the isolate from the breast or colon cancer urinary protein that did not bind to the anti-NHS and Protein A affinity column is shown in Fig. 1. Both isolates show 2 bands at a mol. wt of ~25,000 and 3 bands at

TABLE V.—*Breast cancer TA isolated from urinary protein by anti-breast-TA affinity chromatography and further purified by affinity chromatography with anti-NHS and protein A*

| Preincubation of donor leucocytes | | Donor diagnosis | NAI before incubation | Total protein concentration of blocking material (mg/l) | NAI after blocking |
|---|---|---|---|---|---|
| Bound samples from anti-breast-TA affinity column: Affinity column of anti-NHS and protein A fractions of: | | | | | |
| Metastatic breast ca | | | | | |
| Unbound | Prep. 1 | Breast ca A | 36 | 50 | 0 |
| | Prep. 2 | B | 35 | 50 | 18 |
| | | C | 44 | 1·0 | 12 |
| | | | | 0·5 | 17 |
| | | | | 0·1 | 92 |
| | | | | 0·01 | 74 |
| Metastatic colon ca | | | | | |
| Unbound | | A | 36 | 50 | 33 |
| Normal subjects | | | | | |
| Unbound | | B | 35 | 100 | 43 |
| Metastatic breast ca | | | | | |
| Unbound | | Malignant melanoma | 38 | 50 | 57 |
| Unbound | | Colon ca | 61 | 50 | 78 |

~50,000. However, the isolate from the breast-cancer urinary protein has an intense band at a mol. wt of ~12,000, in contrast to the isolate from the colon-cancer urinary protein, and a unique band at ~38,000 (Fig. 1).

The isolates of the breast-and colon-cancer urinary protein from the anti-NHS affinity column were radiolabelled with $^{125}I$ and run on the SDS slab gels. Fig. 2 shows the autoradiographs of the isolate from the breast-cancer urinary protein before and after incubation and clearing with Protein A on fixed bacteria (Kessler, 1975) and the isolate from the colon cancer urinary protein. The isolate from the breast-cancer urinary protein has a unique band at a mol. wt of ~38,000. The band at ~12,000 mol. wt is more intense in the isolate from breast than colon cancer urinary protein. In comparison, Fig. 2 shows an autoradiograph of papain-soluble breast-cancer membrane material purified by a horse anti-human-$\beta_2$ microglobulin affinity column (Thomson *et al.*, 1976, 1979*b*). This material also blocked specifically the LAI reactivity of leucocytes from breast cancer patients and did not alter that from patients with colon cancer or melanoma.

The anti-NHS and Protein A affinity column removed some of the protein contaminates from the isolates recovered from the anti-breast-TA affinity column with a 2-fold increase in specific activity of the breast TA. However, on SDS PAGE a number of intense, common bands remained in the materials with and without breast-TA activity.

### *Affinity chromatography with anti-nonspecific sera*

Although the TAs in the urine of patients with metastatic cancer could be isolated by an affinity column of tumour-specific IgG, the isolates were clearly contaminated by other species of urinary protein. The anti-NHS and Protein A affinity column did not remove enough of the contaminants from the isolates, so another approach was made.

Antiserum to the nonspecific proteins eluted from the anti-breast affinity column

FIG. 1.—SDS PAGE analysis of the bound and eluted isolates of urinary protein from the anti-breast-TA affinity column that were then passed through the anti-NHS and Protein-A affinity column. A, mol. wt standards; B, urinary protein from patient with metastatic colon cancer; C, urinary protein from patient with metastatic breast cancer. Triangles point to bands in the isolate from breast urinary protein but which are either absent or in minimal amounts in the isolate from colon urinary protein.

FIG. 2.—SDS PAGE analysis (autoradiograms) of $^{125}$I-labelled isolates of the following: A, papain-soluble material from the membranes of breast cancer that were bound and eluted from a horse anti-human-$\beta_2$-microglobulin affinity column; B, bound and eluted isolate of urinary protein from a patient with metastatic breast cancer from the anti-breast-TA affinity column that was passed through the anti-NHS and Protein-A affinity column; C, same material cleared with Protein A of whole fixed Staphylococcus; D, bound and eluted isolate of urinary protein from a patient with metastatic colon cancer from the anti-breast-TA affinity column that was passed through the anti-NHS and Protein-A affinity column.

was prepared as described in Materials and Methods. With this antiserum an anti-nonspecific affinity column was prepared. The possibility that the antiserum might react with the TA in the urine was considered.

The bound and eluted samples of urinary protein from patients with metastatic breast or colon cancer from the anti-breast-TA affinity column were applied to the anti-nonspecific affinity column. Table VI shows that the unbound isolate from urinary protein from patients with metastatic breast cancer negated the LAI of leucocytes from patients with breast cancer, whereas the unbound isolate from the urinary protein of patients with metastatic colon cancer did not block. In contrast, LAI$^+$ leucocytes from patients with colon cancer reacted in the presence of the unbound isolates from the breast- and colon-cancer urinary protein (Table VI). Similarly, the bound and eluted urinary protein from the anti-colon-TA affinity column were applied to the anti-nonspecific affinity column. Table VI shows that the unbound isolate from colon urinary protein nullified LAI in an immunologically specific manner. The anti-nonspecific affinity column increased the TA-specific activity about 5-fold (Tables I and II).

Fifty-$\mu$g samples of the 4 isolates from the anti-breast-TA and anti-colon-TA affinity column were labelled with $^{125}$I. The $^{125}$I-labelled isolates were applied separately to the anti-nonspecific affinity

TABLE VI.—*Breast- or colon-cancer TA isolated from urinary protein by anti-breast-TA or anti-colon-TA affinity chromatography and further purified by anti-nonspecific affinity chromatography*

| Preincubation of donor leucocytes | Donor diagnosis | NAI before incubation | Total protein concentration of blocking material (mg/l) | NAI after blocking |
|---|---|---|---|---|
| Bound samples from anti-breast-TA affinity column: | | | | |
| Affinity column of anti-nonspecific: | | | | |
| Unbound fractions of: | | | | |
| Metastatic breast ca | | | | |
| Prep. 1 | Breast ca A | 77 | 10 | −15 |
| | B | 108 | 1·0 | −28 |
| | | | 0·5 | −16 |
| | | | 0·1 | −22 |
| | | | 0·05 | 45 |
| | | 77 | 0·05 | 35 |
| Metastatic colon ca | | | 25 | 125 |
| Metastatic breast ca | Colon ca | 38 | 25 | 87 |
| Metastatic colon ca | | 60 | 25 | 41 |
| Bound samples from anti-colon-TA affinity column: | | | | |
| Affinity column of anti-nonspecific: | | | | |
| Unbound fractions of: | | | | |
| Metastatic colon ca | | | | |
| Prep. 1 | Colon ca | 41 | 10 | −3 |
| | | | 1·0 | 2 |
| | | | 0·1 | 16 |
| | | | 0·05 | 48 |
| Prep. 2 | | 46 | 10 | −14 |
| | | | 1·0 | 15 |
| | | | 0·5 | 12 |
| | | | 0·2 | 24 |
| | | | 0·1 | 40 |
| Metastatic colon ca | Breast ca | 80 | 25 | 67 |
| Metastatic breast ca | | | 25 | 66 |

column and the unbound material was collected and concentrated. The $^{125}$I-labelled isolates were electrophoresed on SDS gels and the patterns were autoradiographed.

The isolate from the breast urinary protein from the anti-breast TA affinity column shows a heavy band at ~38,000 mol. wt that is not observed in the isolate from the colon urinary protein from the same affinity column (Fig. 3). After passage through the anti-nonspecific affinity column, the $^{125}$I-labelled isolate from the breast urinary protein continued to show a strong band at a mol. wt of about 38,000. Three finer bands at ~25,000–30,000 mol. wt were seen, and at least one of these bands appeared in the isolate from the colon urinary protein after passage through the anti-nonspecific affinity column. Isolates from both colon and breast urinary protein show a band at ~50,000 mol. wt that was not removed by the anti-nonspecific affinity column.

An autoradiograph of the isolate from the colon-cancer urinary protein from the anti-colon-TA affinity column shows a band at ~40,000 mol. wt (Fig. 4), whereas the isolate from the colon urinary protein from the anti-breast-TA affinity column lacked a band at this mol. wt (Figs 2 and 3). After passage through the anti-nonspecific affinity column, the isolate from the colon urinary protein from the anti-colon-TA affinity column continued to have a heavy band at ~40,000 mol. wt.

FIG. 3.—SDS PAGE analysis (autoradiograms) of $^{125}$I-labelled isolate containing breast TA activity and control material. A, urinary protein from patient with metastatic breast cancer bound and eluted from the anti-breast-TA-affinity column; B, same material applied and unbound on the anti-nonspecific affinity column; C, urinary protein from patient with metastatic colon cancer bound and eluted from the anti-breast-TA affinity column; D, same material applied and unbound from the anti-nonspecific affinity column.

FIG. 4.—SDS PAGE autoradiograms of $^{125}$I-labelled material from an isolate containing colon-TA activity. A, urinary protein from patient with metastatic colon cancer unbound from the anti-breast-TA affinity column and bound and eluted from the anti-colon-TA affinity column; B, same material applied and unbound on the anti-nonspecific affinity column.

The heavy band at 25,000 mol. wt was removed, although 4 fine bands remained at 25,000–30,000 mol. wt. A faint band at ~12,000 mol. wt is also present.

In comparison, an autoradiograph of the isolate from the breast urinary protein from the anti-colon TA affinity column is shown in Fig. 5. A heavy band is present at ~25,000 mol. wt with faint bands at ~50,000 and 12,000 mol. wt. However, the isolate from the breast urinary protein from the anti-colon-TA affinity column lacked a band at ~40,000 vol. wt (Fig. 5). After passage of the breast urinary protein from the anti-colon-TA affinity column through the anti-nonspecific affinity column, no band at 40,000 mol. wt was seen, although a band at 50,000 mol. wt and a faint band at ~25,000 mol. wt remained.

### DISCUSSION

Since the TA is denatured by SDS PAGE, there is no way to determine which, if any, of the bands visualized in the gels carries the TA epitope. Previously we have shown that human TAs papain-solubilized from the membranes of hepatoma, malignant melanoma, breast and colon cancer were linked to $\beta_2$-microglobulin (Thomson *et al.*, 1976, 1979*b*; Thomson, 1979). In this study, the material with specific TA activity had a 12,000-mol.-wt subunit which became fainter with purification, which suggests that the association between $\beta_2$-microglobulin and the molecule which carries the TA epitope was partially ruptured during isolation. Histocompatibility antigens isolated from urine are reported to show dissociation of the heavy chain and $\beta_2$-microglobulin (Bernier *et al.*, 1974).

Human cancers express organ-type-specific neoantigens which are immunogenic to the host bearing the cancer. The same antigens exist in some premalignant lesions such as colon adenomas (Tataryn

FIG. 5.—SDS PAGE autoradiogram of urinary protein from patient with metastatic breast cancer unbound from the anti-breast-TA affinity column and bound and eluted from the anti-colon-TA affinity column.

*et al.*, 1979) and in tissues of organs that display dysplastic changes of epithelium which may or may not be premalignant, such as papillomatosis and fibrocystic disease of the breast (Flores *et al.*, 1977; Lopez *et al.*, 1978; O'Connor *et al.*, 1978; Thomson *et al.*, 1979; Sanner *et al.*, 1979) and chronic atrophic gastritis (Tataryn *et al.*, 1979). The epitope of the organ-specific neoantigen has not been detected in the tissues of normal organs (Grosser & Thomson, 1975; O'Connor *et al.*, 1978; Thomson *et al.*, 1979*b*; Tataryn *et al.*, 1979). The organ-specific neoantigen is probably not synthesized *de novo*, but the mutational process involved in the genesis of the cancer or dysplastic cell may induce some rearrangement of a cell-surface protein of the normal cell. The nature of the change in the cell-surface protein is unknown, but might represent exposure of cryptic sites or structural changes. The question which cell-surface protein has an organ-specific neoantigen epitope that becomes immunogenic will probably not be answered until the molecule is purified and characterized.

This work was supported by grants from the Medical Research Council of Canada, The Cancer Research Society Inc. of Montreal and The National Cancer Institute of Canada.

### REFERENCES

BALDWIN, R. W. & EMBLETON, M. J. (1970) Detection and isolation of tumour-specific antigen associated with a spontaneously arising rat mammary carcinoma. *Int. J. Cancer*, **6**, 373.

BERNIER, I., DAUTIGNY, A., COLOMBANI, J. & JOLLES, P. (1974) Purification of HL-A antigens from the urine of diseased and healthy individuals. *FEBS Lett.*, **45**, 308.

BURGER, D. R., VANDENBARK, A. A., FINKE, P. & 6 others (1977) Assessment of reactivity to tumor extracts by leukocyte adherence inhibition and dermal testing. *J. Natl Cancer Inst.*, **59**, 317.

CAMBIASO, C. L., GOFFINET, A., VAERMAN, J. P. & HEREMANS, J. F. (1975) Glutaraldehyde-activated aminohexyl-derivative of Sepharose 4B as a new versatile immunoadsorbent. *Immunochemistry*, **12**, 273.

FLORES, M., MARTI, J. H., GROSSER, N., MACFARLANE, J. K. & THOMSON, D. M. P. (1977) An overview: antitumor immunity in breast cancer assayed by tube leucocyte adherence inhibition. *Cancer*, **39**, 494.

GROSSER, N., MARTI, J. H., PROCTOR, J. W. & THOMSON, D. M. P. (1976) Tube leukocyte adherence inhibition assay for the detection of anti-tumour immunity: I. Monocyte is the reactive cell. *Int. J. Cancer*, **18**, 39.

GROSSER, N. & THOMSON, D. M. P. (1975) Cell-mediated antitumour immunity in breast cancer patients evaluated by antigen-induced leucocyte adherence inhibition in test tubes. *Cancer Res.*, **35**, 2571.

GROSSER, N. & THOMSON, D. M. P. (1976) Tube leukocyte (monocyte) adherence inhibition assay for the detection of anti-tumour immunity: III. "Blockade" of monocyte reactivity by excess free antigen and immune complexes in advanced cancer patients. *Int. J. Cancer*, **18**, 58.

HALLIDAY, W. S. & MILLER, S. (1972) Leukocyte adherence inhibition: a simple test for cell-mediated tumor immunity and serum blocking factors. *Int. J. Cancer*, **9**, 477.

HOLAN, V., HASEK, M., BUBENIK, J. & JITKA, C. H. (1974) Antigen-mediated macrophage adherence inhibition. *Cell Immunol.*, **13**, 107.

KESSLER, S. W. (1975) Rapid isolation of antigens from cells with a staphylococcal Protein A-antibody absorbent: Parameters of the interaction of antibody–antigen complexes with Protein A. *J. Immunol.*, **115**, 1617.

LAEMMLI, U. K. (1970) Cleavage of structural proteins during the assembly of the head of bacteriophage T4. *Nature*, **227**, 680.

LEVESON, S. H., HOWELL, J. H., HOLYOKE, E. D. & GOLDROSEN, M. H. (1977) Leukocyte adherence inhibition: an automated microassay demonstrating specific antigen recognition and blocking activity in two murine tumor systems. *J. Immunol. Methods*, **17**, 153.

LOPEZ, M., O'CONNOR, R., MACFARLANE, J. K. & THOMSON, D. M. P. (1978) The natural history of antitumour immunity in human breast cancer assayed by tube leucocyte adherence inhibition. *Br. J. Cancer*, **38**, 660.

LOPEZ, M. & THOMSON, D. M. P. (1977) Isolation of breast cancer tumour antigen from serum and urine. *Int. J. Cancer*, **20**, 834.

MALUISH, A. E. & HALLIDAY, W. J. (1975) Quantitation of anti-tumour cell-mediated immunity by a lymphokine-dependent reaction using small volumes of blood. *Cell Immunol.*, **17**, 131.

MARTI, J. H., GROSSER, N. & THOMSON, D. M. P. (1976) Tube leukocyte adherence inhibition assay for the detection of anti-tumour immunity: II. Monocyte reacts with tumour antigen *via* cytophilic anti-tumour antibody. *Int. J. Cancer*, **18**, 48.

O'CONNOR, R., MACFARLANE, J. K., MURRAY, D. & THOMSON, D. M. P. (1978) A study of false positive and negative responses in the tube leucocyte adherence inhibition (tube LAI) assay. *Br. J. Cancer*, **38**, 674

POWELL, A. E., SLOSS, A. M., SMITH, R. N., MAKLEY, J. T. & HUBAY, C. E. (1975) Specific responsiveness of leukocytes to soluble extracts of human tumours. *Int. J. Cancer*, **16**, 905.

REIF, A. E. (1969) Batch preparation of rabbit G-globulin with DEAE cellulose. *Immunochemistry*, **6**, 723.

RUSSO, A. J., DOUGLASS, H. O., JR, LEVESON & 5 others (1978) Evaluation of the microleukocyte adherence inhibition assay as an immunodiagnostic test for pancreatic cancer. *Cancer Res.*, **38**, 2023.

SANDERSON, A. R. (1977) HLA "help" for human B2-microglobulin across species barriers. *Nature*, **269**, 414.

SANNER, T., BRENNHOVD, I., CHRISTENSEN, I., JORGENSEN, O. & KVALOY, S. (1979) Cellular antitumor immune response in women with risk factors for breast cancer. *Cancer Res.*, **39**, 654.

SHANI, A., RITTS, R. E., JR, THYNNE, G. S., WEILAND, L. H., SILVERS, A., MOERTEL, C. G. & GO, V. L. W. (1978) A prospective evaluation of the leukocyte adherence inhibition test in colorectal cancer and its correlation with carcinoembryonic antigen levels. *Int. J. Cancer*, **22**, 113.

TATARYN, D. N., MACFARLANE, J. K., MURRAY, D. & THOMSON, D. M. P. (1979) Tube leukocyte adherence inhibition (LAI) assay in gastrointestinal (GIT) cancer. *Cancer*, **43**, 898.

TATARYN, D. N., MACFARLANE, J. K. & THOMSON, D. M. P. (1978) Leucocyte adherence inhibition for detecting specific tumour immunity in early pancreatic cancer. *Lancet*, i, 1020.

THOMSON, D. M. P. (1979) Demonstration of tube leukocyte adherence inhibition assay with coded samples of blood. *Cancer Res.*, **39**, 627.

THOMSON, D. M. P. & ALEXANDER, P. (1973) A cross-reacting embryonic antigen in the membrane of rat sarcoma cells which is immunogenic in the syngeneic host. *Br. J. Cancer*, **27**, 35.

THOMSON, D. M. P., GOLD, P., FREEDMAN, S. O. & SHUSTER, J. (1976) The isolation and characterization of tumor-specific antigens of rodent and human tumors. *Cancer Res.*, **36**, 3518.

THOMSON, D. M. P., RAUCH, J. E., WEATHERHEAD, J. C. & 5 others (1978) Isolation of human tumour-specific antigens associated with $\beta_2$ microglobulin. *Br. J. Cancer*, **37**, 753.

THOMSON, D. M. P., SELLENS, V., ECCLES, S. & ALEXANDER, P. (1973) Radio-immunoassay of tumour specific transplantation antigen of a chemically induced rat sarcomata: circulating soluble tumour antigen in tumour-bearers. *Br. J. Cancer*, **28**, 377.

THOMSON, D. M. P., TATARYN, D. N., LOPEZ, M., SCHWARTZ, R. & MACFARLANE, J. K. (1979*a*) Human tumor-specific immunity assayed by a computerized tube leukocyte adherence inhibition. *Cancer Res.*, **39**, 638.

THOMSON, D. M. P., TATARYN, D. N., O'CONNOR, R. & 4 others (1979*b*) Evidence for the expression of human tumor-specific antigens associated with $\beta_2$ microglobulin in human cancer and in some colon adenomas and benign breast lesions. *Cancer Res.*, **39**, 604.

ZOLLER, M. & MATZKU, S. (1976) Antigen and antibody purification by immunoadsorption: elimination of non-biospecifically bound proteins. *J. Immunol. Methods*, **11**, 287.